# Coincidence of Large Adrenal Cyst and Prominent Hyporeninemic Hyperaldosteronism

**DOI:** 10.1155/2021/8860498

**Published:** 2021-02-20

**Authors:** Takaaki Sakaue, Yosuke Okuno, Kosuke Mukai, Shingo Fujita, Junji Kozawa, Hitoshi Nishizawa, Taka-Aki Matsuoka, Hiromi Iwahashi, Maeda Norikazu, Yuto Yamazaki, Hironobu Sasano, Michio Otsuki, Iichiro Shimomura

**Affiliations:** ^1^Department of Metabolic Medicine, Osaka University Graduate School of Medicine, Osaka 565-0871, Japan; ^2^Department of Diabetes Care Medicine, Osaka University Graduate School of Medicine, Osaka 565-0871, Japan; ^3^Department of Metabolism and Atherosclerosis, Osaka University Graduate School of Medicine, Osaka 565-0871, Japan; ^4^Department of Pathology, Tohoku University Graduate School of Medicine, Sendai 980-8574, Japan

## Abstract

A 67-year-old Japanese woman who had end-stage renal disease was referred to our hospital for kidney transplantation. Abdominal CT revealed a large adrenal mass with inhomogeneity. She had a history of hospitalization for stroke and heart failure and exhibited prominent hyporeninemic hyperaldosteronism. Histological examination of the resected tumor with anti-CYP11B2 antibody indicated that she had a vascular endothelial cyst with primary aldosteronism (PA) due to multiple adrenocortical micronodules. This report implicates the pathological interaction between adrenal vascular cysts and PA-mediated vascular damage of the adrenal vein.

## 1. Introduction

Adrenal cysts are rare and form a heterogeneous group of lesions. Their incidence in autopsy has been reported to be 0.064–0.18% [1]. However, the frequency with which they are disclosed appears to be increasing because of improved radiological imaging techniques such as computed tomography (CT) and magnetic resonance imaging (MRI) [2]. Adrenal vascular endothelial cysts are one of the subtypes of adrenal cysts and associated with vascular damage [3]. Although aldosterone is directly involved in such vascular damage, coincidence of these two disorders was scarcely reported.

Etiologically, hypertension induced by excess aldosterone secretion features more prominent renal damage than essential hypertension [4]. When not treated appropriately, it eventually leads to end-stage renal disease (ESRD). Under these circumstances, endocrinological examinations are less valuable because of aberrant hormone metabolism and the risk of confirmation tests.

We herein present a case of combination of large adrenal endothelial cyst and prominent hyporeninemic hyperaldosteronism with ESRD where immunohistological examination was useful.

## 2. Case Presentation

**Figure 1:**
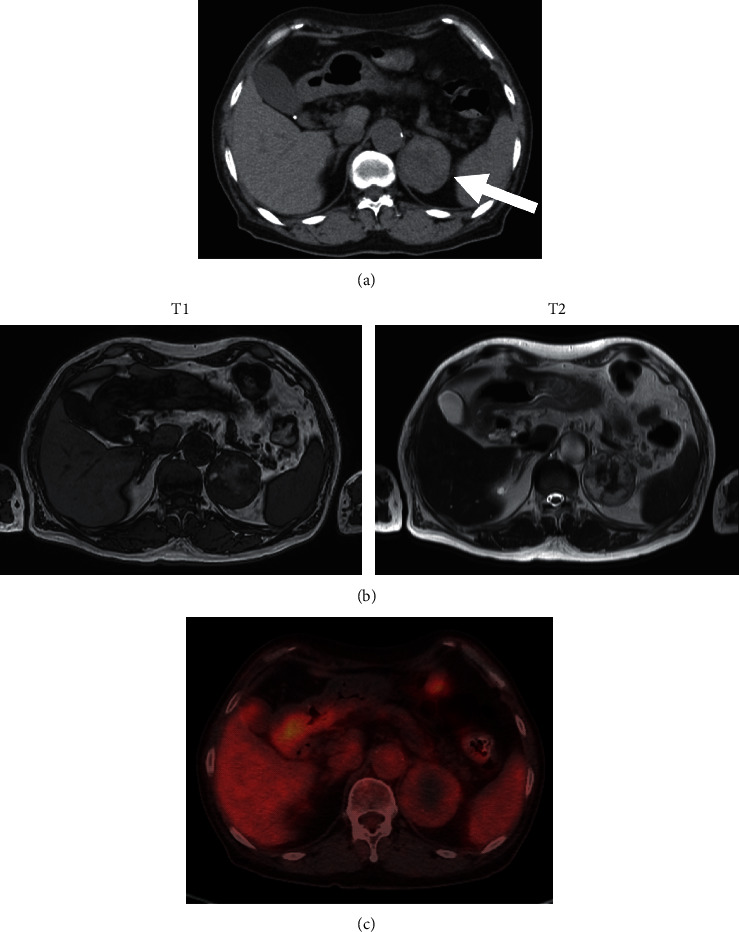
(a) Abdominal plain CT. Arrow: left adrenal mass (52 mm × 50 mm). (b) T1-weighed (left) and T2-weighed (right) images of plain MRI. (c) PET-CT.

A 67-year-old Japanese woman was referred to our hospital for renal replacement therapy and kidney transplantation. She had been diagnosed as hypertension at her late 20s, but blood pressure was poorly controlled despite treatment with antihypertensive drugs without an aldosterone antagonist. She had a history of hospitalization for stroke at 48 years old and heart failure at 53 years old. She was administrated with warfarin for atrial fibrillation. She was diagnosed as diabetes at 62 years old, but her glycemia was well-controlled with linagliptin. Her renal function had gradually impaired, most likely due to nephrosclerosis. In April 2018, her estimated glomerular filtration rate (eGFR) was 7 ml/min/1.73 m^2^. During the checkup for kidney transplantation, abdominal CT revealed a left adrenal mass, which was 5 cm in diameter with inhomogeneity of density and smooth borders (Figure 1(a)). She was admitted in August 2018 for endocrinological examination.

On admission, her blood pressure was 133/89 mmHg with 5 mg amlodipine, 60 mg azosemide, and 0.625 mg bisoprolol fumarate. She had no Cushingoid appearances. The plasma parameters are shown in Table 1. Her serum potassium level was at the lower limit of normal despite ESRD. She exhibited prominent hyporeninemic hyperaldosteronism. The plasma aldosterone concentration (PAC) was 514.9 pg/ml, and plasma renin activity (PRA) was <0.2 ng/ml/hr, but we could not perform saline infusion test nor captopril challenge test in afraid of deteriorating renal function and heart failure. The plasma ACTH level and serum cortisol level were within normal limits. The serum dehydroepiandrosterone-sulfate (DHEA-S) level was below the lower limit of normal. The midnight serum cortisol level was above 5 *μ*g/dl. Overnight 1 mg dexamethasone suppression test could not suppress the serum cortisol level below 3 *μ*g/dl. Twenty-four-hour urinary excretion of cortisol was below the detection limit. Twenty-four-hour urinary excretion of catecholamines and their metabolites did not exceed normal limits. The serum testosterone and estradiol levels were within normal limits.

High-intensity in T1-weighted images (T1WI) and low-intensity in T2WI of abdominal MRI suggested bleeding in the adrenal mass (Figure 1(b)). ^123^I-metaiodobenzylguanidine (MIBG) was not uptaken into the adrenal mass (data not shown). On the positron emission tomography with fluorodeoxyglucose (FDG-PET), the mass had activity less than that of the liver (Figure 1(c)). Considering the size above 4 cm and bleeding inside, the tumor was laparoscopically resected for suspected malignancy. After surgery, PAC, PRA, plasma ACTH, and serum cortisol remained almost unchanged, while blood pressure was decreased to 108/70 mmHg and serum potassium level was increased to 4.4 mEq/L (Table 1).

**Figure 2:**
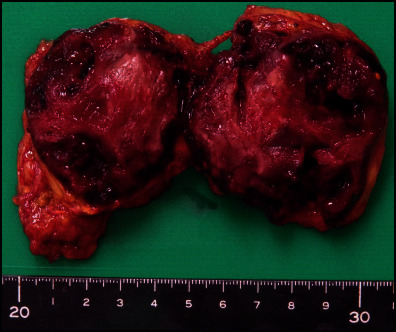
Gross inspection of the resected tumor.

**Figure 3:**
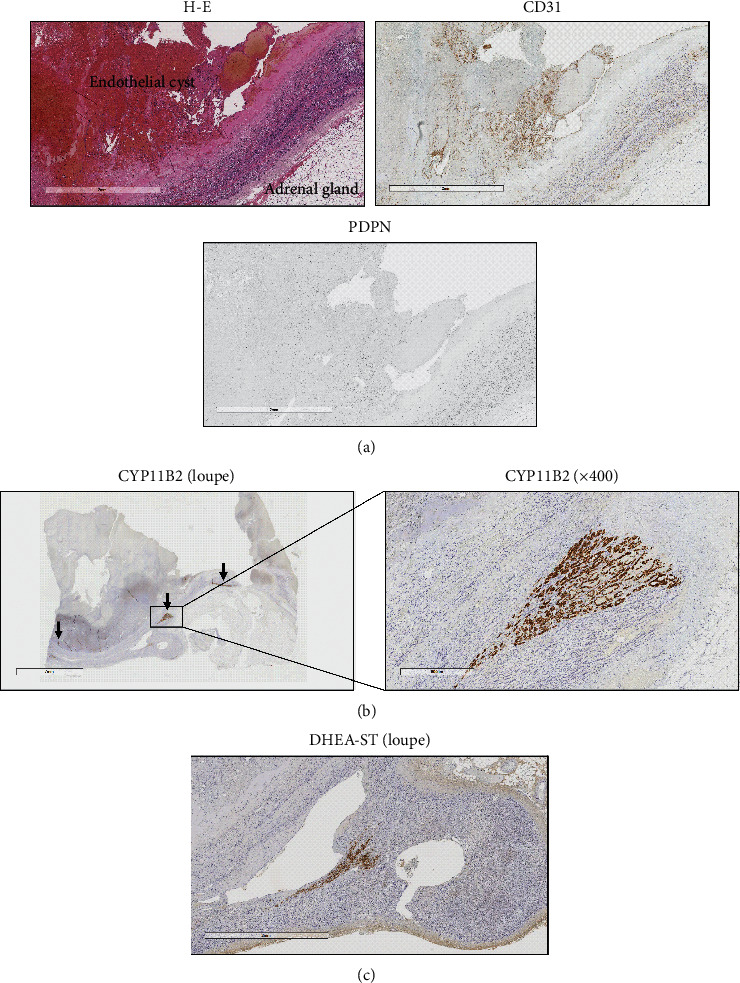
(a) H-E staining (H-E) immunostaining with anti-CD31 antibody (CD31) or anti-PDPN (D2-40) antibody (PDPN) of the resected tumor (magnification: x200). (b) Immunostaining of the resected tumor with anti-CD11B2 antibody. Left: loupe, right: x400 magnification. Arrow: CYP11B2-positive regions. (c) Immunostaining with anti-DHEA-ST antibody of the adjacent adrenal gland (magnification: x20).

On gross inspection, the tumor exhibited multiple nodular solid lesions with prominent bleeding (Figure 2). The blood tumor was an adrenal vascular endothelial cyst surrounded by CD31-positive/PDPN-negative endothelial lining and papillary hyperplasia (Figure 3(a)). There was no arteriovenous malformation. As a possible source of hyperaldosteronism, CYP11B2-positive multiple adrenocortical micronodules (MN) surrounded by hyperplastic zona glomerulosa without diffuse overexpression of CYP11B2 were identified (Figure 3(b)). The expression of dehydroepiandrosterone sulfotransferase (DHEA-ST) in zona reticularis was normal (Figure 3(c)).

## 3. Discussion

In this paper, we report the unique case of prominent hyporeninemic hyperaldosteronism with a large vascular endothelial cyst. The coincidence of hyporeninemic hyperaldosteronism and adrenal cyst was reported only in one case report, where primary aldosteronism (PA) by adrenal adenoma was combined with ipsilateral pseudocyst [5].

The cause of adrenal vascular endothelial cysts and pseudocysts is not clear, but they are considered to originate from prior hemorrhage from pre-existing vascular hamartoma or adrenal vein [3, 6]. As aldosterone is known to be directly involved in vascular damage [7] and hemodynamics within the tissue [8], it might attribute to such adrenal hemorrhages. For example, intra-adrenal veins are considered to be uniquely fragile in patients with PA, from the fact that extremely high rate of intra-adrenal hemorrhage was experienced specifically in adrenal venography of PA [9]. As a possible mechanism, mineralocorticoid receptor was expressed in vascular smooth muscle cells (VSMC) [10] and direct mineralocorticoid effect on vascular tone and contractility has been suggested [11–13]. Aldosterone also stimulates fibroblast-mediated fibrosis in a vascular matrix [14–16]. PA not only increases intravascular volume in the whole body [17] but also decreases local vascular resistance, at least in the kidney [8], which may increase the flow volume in the tissue. Analogically, PA might also increase the flow volume of the adrenal vein and make it prone to hemorrhage. In this case, hemorrhagic diathesis by warfarin would be attributed to adrenal hemorrhages. ESRD may also be attributed to vascular cysts, as congestion or hemorrhage was frequently observed in the adrenal of patients with ESRD in an autopsy analysis [18].

In patients with ESRD, it is difficult to evaluate autonomous aldosterone secretion. The aldosterone-to-renin ratio (ARR) is elevated in chronic renal disease [19], and saline infusion test or captopril challenge test need caution as they might exaggerate the renal impairment. In this case, we could not endocrinologically diagnose but strongly suspected PA from poorly controlled hypertension, multiple PA-related complications, and low serum potassium despite ESRD, together with prominent hyporeninemic hyperaldosteronism. Identification of CYP11B2-positive lesions surrounded by hyperplastic zona glomerulosa further supported this speculation [20]. In this case, as serum potassium level and blood pressure were improved after surgery, significant amount of aldosterone may be produced by the same side of adrenal. Although hyporeninemic hyperaldosteronism still existed after removal of the left adrenal containing CYP11B-positive lesions, it was not surprising as the majority of MN is bilateral [21]. If this case was PA, aldosterone concentration would be much higher in the adrenal vein than in the blood, which further accelerated the formation of adrenal endothelial cyst.

It is also challenging to evaluate autonomous cortisol secretion in ESRD. The morning plasma cortisol is higher, and 1 mg dexamethasone fails to suppress plasma total cortisol in patients with chronic renal failure [22]. The serum DHEA-S level is decreased in hemodialysis patients [23]. In this case, the possibility of coexisting subclinical Cushing syndrome could not be ruled out by dexamethasone suppression test or midnight serum cortisol, but was unlikely from the normal expression of DHEA-ST in zona reticularis. We assumed that the patient was in the pseudo-Cushing state caused by ESRD.

This case implicated the pathological interaction between adrenal vascular cyst and aldosterone-mediated vascular damage or hemodynamics of adrenal vein and showed the usefulness of immunohistochemistry to understand the endocrinological state in ESRD.

LH : luteinizing hormone, FSH : follicle-stimulating hormone, PRL : prolactin, ACTH : adrenocorticotropic hormone, DHEA-S : dehydroepiandrosterone-sulfate, TSH : thyroid stimulating hormone, FT3 : free triiodothyronine, FT4 : free thyroxine, PRA : plasma renin activity, PAC : plasma aldosterone concentration, and U-VMA : urinary vanillyl mandelic acid.

## Figures and Tables

**Table 1:** Plasma parameters.

Blood sample: on admission					
WBC	4.52 × 10^3^	/*μ*L	RBC	4.41 × 10^6^	/*μ*L
Hb	11.9	g/dL	Plt	187 × 10^3^	/*μ*L
Creatinine	6.61	mg/dL	eGFR	5.4	ml/min/1.73 m^2^
Na	144	mEq/L	K	3.7	mEq/L
PRA	≦0.2	ng/mL/hr	PAC	514.9	pg/mL
ACTH	25	pg/mL	Cortisol	14.5	*μ*g/dL
DHEA-S	25	*μ*g/dL	Estradiol	<14.0	ng/mL
Testosterone	0.24	ng/mL	HbA1c	5.6	%

Urine sample					
U-adrenaline	1.08	*μ*g/day	U-VMA	2.16	mg/day
U-noradrenaline	55.26	*μ*g/day	U-metanephrine	0.036	mg/day
U-dopamine	100.8	*μ*g/day	U-normetanephrine	0.126	mg/day
U-cortisol	Below the detection limit				

Diurnal profile of ACTH and cortisol					
Time	6 : 00	23 : 00			
ACTH (pg/mL)	23	7			
Cortisol (*μ*g/dL)	16.4	6.9			

Low-dose dexamethasone suppression test					
	After 1 mg
ACTH (pg/mL)	2				
Cortisol (*μ*g/dL)	4.5				

Blood sample: one month after surgery					
K	4.4	mEq/L	eGFR	5.1	ml/min/1.73 m^2^
PRA	≦0.2	ng/mL/hr	PAC	533.6	pg/mL
ACTH	31	pg/mL	Cortisol	13.5	*μ*g/dL
DHEA-S	3.8	*μ*g/dL			
